# Misalignment analysis of WPT level 3/Z2-class of CirPT with DDPR and CirPR for EVs stationary charging

**DOI:** 10.1038/s41598-024-76381-2

**Published:** 2024-11-05

**Authors:** Mahmoud M. Elymany, Ahmed A. S. Mohamed, Ahmed A. Shaier, Mohamed A. Enany, Hamid Metwally, Sameh I. Selem

**Affiliations:** 1 Electrical Power and Machines Department, Faculty of Engineering, Zagazig University, Zagazig, 44519 Egypt; 2 Eaton Research Labs, Eaton Corporate, Golden, CO USA

**Keywords:** Misalignment scenarios, CirPT—CirPR configuration, CirPT-DDPR configuration, Interoperability, Electric Vehicle (EV), Electrical and electronic engineering, Energy storage, Renewable energy

## Abstract

Future inductive charging ports must possess the capability to charge any electric vehicle (EV), irrespective of the specific coil architecture it is equipped with. This study examines the misalignment scenarios of the global circular pad at transmitter side (CirPT) with circular receiver pad (CirPR) and a double-D receiver pad (DDPR). The CirPT, CirPR, and DDPR configurations for WPT3 (11.1 kW) with ground clearance meeting the Z2-class specifications and above ground surface installation are built by utilizing circuit analysis and 3D-finite element simulations, as outlined by the Society of Automotive Engineering (SAE) J2954 standard. The simulated designs are employed to determine the frequency (*f*) and the compensating network components (CNCs) required to achieve optimal power transfer efficiency while maintaining nominal power levels. The analysis of misalignment scenarios involves examining various performance factors, including coupling coefficient (*k*), transmission power (*P*_*o*_), efficiency (*η*), and leakage electromagnetic fields (EMFs). These factors are assessed under conditions of ideal alignment, as well as various linear and angular misalignments within the inductive charging system. The results demonstrate that both the CirPR and DDPR configurations can successfully interface with the CirPT to provide the required *P*_*o*_ to the EV battery with commendable efficiency. In perfect alignment, the efficiencies are 95.10% for the CirPT-CirPR model and 91.60% for the CirPT-DDPR model. In maximum misalignment, the efficiencies are 87.10% for the CirPT-CirPR model and 89.50% for the CirPT-DDPR model, all exceeding the acceptable threshold of 80%.

## Introduction

In recent times, electrification has emerged as a significant revolution within the transportation sector, aimed at enhancing safety, efficiency, and environmental sustainability. Utilizing an electric vehicle (EV) result in reduced reliance on fossil fuels, decreased emissions of greenhouse gases, lowered operating expenses, and enhanced driving satisfaction. However, significant obstacles hindering the widespread adoption of EVs include concerns about range anxiety, lengthy recharging durations, and the availability of charging infrastructure. Inductive charging technology stands as a promising solution to address these challenges. It enables electric vehicles to wirelessly receive electrical energy without the need for connected wires. This system can be integrated into forthcoming charging stations, both in general and special garages, to facilitate static charging. Additionally, it may be deployed along routes to enable EV charging while in motion (dynamic charging)^1^, as exemplified by the FABRIC venture, or while brief pauses (semi-dynamic charging)^2^. Inductive magnetic couplers offer numerous benefits, including automatic operation without requiring driver intervention, safety and convenience in adverse conditions like snow, rain, and dust, and maintenance-free operation due to the elimination of plug-in connections^3^. Additionally, in-motion charging holds the promise of substantially extending vehicle range, eliminating recharging downtime, and reducing reliance on the EV onboard battery^4,5^. In IPT architectures, electromagnetic energy is transported in-between the ground side pad (GSP) and car side pad (CSP) across a broad air gap without any visible contact. This technology finds application across various domains, including medical equipment, portable smart devices, household appliances, and EVs^6^.

**Fig. 1:**
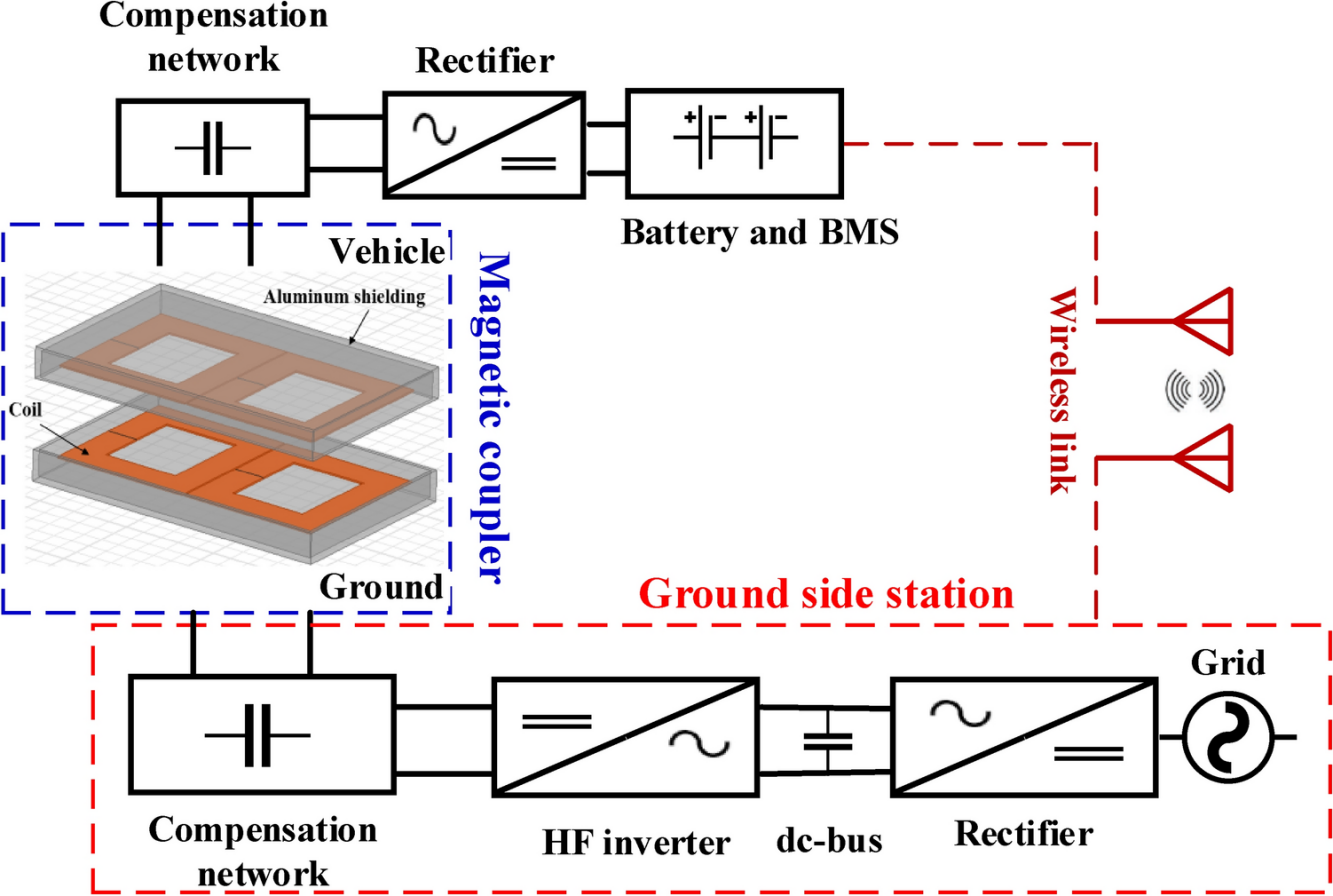
Components of GSP and CSP for typical IPT configuration.

A typical architecture of the magnetic coupler for EVs comprises two separate units: the GSP and the CSP, as illustrated in Fig. 1. The ground-based station is positioned beneath the ground surface and includes components such as a power source, a DC-bus, a GSP rectifier, a CNCs, and an inverter that operates high-frequencies (HF). This station feed the transmitter coil with power. The car-side pad is mounted beneath the EV and comprises a receiver pad, CNCs, and rectifier, which delivers power to the battery (load). IPT configurations typically function at HF fall between 79-kHz and 90-kHz to achieve substantial power transfer capabilities while maintaining a size that is suitable for installation within the vehicle. The CSP talks with the GSP via a communication link for tasks such as authentication, alignment setting, control management, and pay billing.

The magnetic inductive coupler, comprising both GSP and CSP, stands as a critical ingredient within the charging process, tasked with facilitating the transfer of power from the grid to the car. Numerous pad architectures have been introduced in the literature, including non-polarized pads (NPPs) like circular (CirP) and rectangular (RP) ones, where the majority of linked flux travels vertically. Additionally, there are structures that rely on horizontal flux ingredients, which known as polarized pads (PPs) like DDP, DDQP, and bi-polar pads (BP)^7^. SAE J2954 identifies two architectures from a range of pad structures to symbolize each category: CirP for orthogonal flux representation, and DDP for horizontal flux representation^8^. Lately, numerous organizations have shown interest in setting up general inductive charging ports to streamline charging processes of EV and promote the development of safer and more environmentally friendly transportation systems.

A significant hurdle hindering the market adoption of this technique is the issue of interoperability. This entails ensuring that any EV can utilize any charging station, irrespective of its architecture or maker. Consequently, it’s imperative to design a system that enables seamless integration among different architectures, manufacturers, and power levels, even in various cases of misalignment. This is particularly crucial for systems intended for public utilize, like public garages, building, and parking.

Numerous researchers have delved into the study of misalignment, endeavoring to devise topologies that offer interoperability^9,10^. Other research has focused on evaluating how different structures perform when they work in tandem^11–14^. The study described in^9^, explored the misalignment analysis of a 3.3-kW inductive coupler. This analysis involved a bipolar pad serving as the GSP and CirP, DDP, and solenoid pad (SP) acting as CSP^15^. The main factors considered in the investigation included efficiency (*η*), ampere-turns (ATs), and coupling coefficient (*k*). The study in^12^ evaluated the effectiveness of a quadruple pad consisting of four interconnected rectangular coils, comparing it with DDP,RP, and DDQP configurations with a focus on lateral misalignments. An investigation was conducted on an inductive magnetic coupler that transport power level of 3.3-kW, examining a DDP on the GSP and a unipolar coil on the CSP, as discussed in^8,13^. The analysis assessed interoperability by examining factors such as *k*, overall *η*, and the extent of electromagnetic field leakage. This evaluation took into account misalignments in the linear directions, namely X, Y, and Z axes. The study in^14^ took into account the interoperability definition outlined in the IEC 61980-3^16^, to investigate the functionality of DDP and SP acting as GSP paired with two flat pads, each containing a large rectangular coil and two smaller circular coils inside it, serving as CSP. Testing was conducted at a 3-kW power level, focusing on factors like coupling coefficient, overall efficiency, and power transfer performance.

The SAE J2954 specifies that the GSP architecture depend on circular pad design (CirPT) for WPT3 (11.1-kW) will be taken into account for public utilization^17^. This study presents an extensive examination of misalignment concerning the universal ground side pad (GSP) suggested for public application in the SAE J2954^17^. It delves into the functionality of CirPT for WPT3 (11.1-kW) when operating with DDPR and CirPR configurations, considering a ground clearance corresponding to the Z2-class standard. The main contributions of this study include:

- Constructing and modeling 3D finite-element (3D-FEMs) designs in accordance with the guidelines suggested in SAE j2954 (power level of WPT3 (11.1-kW) and ground clearance of Z2-class requirements) for:
  - A circular pad as universal transmitter (CirPT).
  - A circular pad as receiver (CirPR).
  - A DD pad as receiver (DDPR).
- Creating individual circuits for each model using MATLAB Simulink at Z2-class.
- Determining the frequency (*f*) of operation for each model, compensation network components (CNCs), and appropriate values of capacitors and inductors of the filter in both GSP and CSP, to maximize the efficiency and transfer the nominal power.
- Examining the *k*, *P*_*o*_, and *η* in scenarios of ideal alignment, linear misalignment, and rotational misalignment.
- Investigating the safety degree of the proposed configurations (CirPT-CirPR, and CirPT-DDPR) in terms of electric and magnetic fields.

The remainder of the manuscript is structured as follows: The detailed steps for modeling the two magnetic designs are discusses in Sect. “Magnetic charger architecture modeling”, then the CNCs analysis are presented in Sect. “Compensation network components (CNCs)”. In Sect. “Interoperability assessment”, an evaluation of the performance factors assessing interoperability and misalignment scenarios is carried out. In Sect. “Discussion and findings”, the findings of both CirPT-CirPR and CirPT-DDPR configurations are discussed and compared. Finally, the outcomes are summarized in Sect. “Conclusion and future work”.

## Magnetic charger architecture modeling

Owing to the intricate configuration of the magnetic coupler, obtaining precise analytical settlements for electromagnetic flux (EMF) propagation can’t be feasible. Therefore, various numerical methods are employed to analyze the EMF. In this research, the ANSYS Maxwell magneto-static solution is employed to calculate EMFs and estimate magnetic parameters. 3D FEMs are created and evaluated in accordance with the guidelines outlined in the SAE J2954 standard^7^.

### Standard levels of power and air-gaps requirements

Designing a charging system entail ensuring sufficient power delivery to the EV battery within a suitable timeframe. This necessity imposes limitations on the charger’s power output in general, and specifically on inductive charging systems. As per the specifications outlined in SAE J2954, The *P*_*o*_ levels for the magnetic charger of light-duty EVs (LDEVs), along with corresponding ground clearance rage (GCR) are presented in Table 1. These classifications aim to encompass various types and models of LDEVs. The ground clearance signifies the orthogonal separation from the lowest coil surface of the CSP and the ground surface. The orthogonal magnetic separation, also known as “airgap”, between the bottom of CSP and the top of GSP relies on the installation of GSP, whether it is positioned above ground surface, flush with ground surface, or beneath ground surface. In anticipation of future general charging ports, it is anticipated that a flush installation method will be employed. This approach aims to minimize the airgap and streamline access to the GSP for repair and rehabilitation purposes. This research examines the effectiveness of the global circular pad (CirPT) when paired with various receiver pads (CirPR and DDPR), considering WPT level 3 and adhering to the maximum threshold of the Z2-class (210 mm).

**Table 1:** SAE j2954 recommendations concerning *P*_*o*_ levels and Z-class requirements.

Power levels for light-duty EVs (kVA)				Ground clearance range (GCR) (mm)			Actual air gap’ (Cases of GSP)		
WPT1	WPT2	WPT3	WPT4	Z1-class	Z2-class	Z3-class	Under ground	Flush ground	Above ground
3.7	7.7	11.1	22	100–150	140–210	170–250	< GCR	= GCR	> GCR

### Modeling of CirPT, CirPR, and DDPR for WPT level 3/Z2-class

**Fig. 2:**
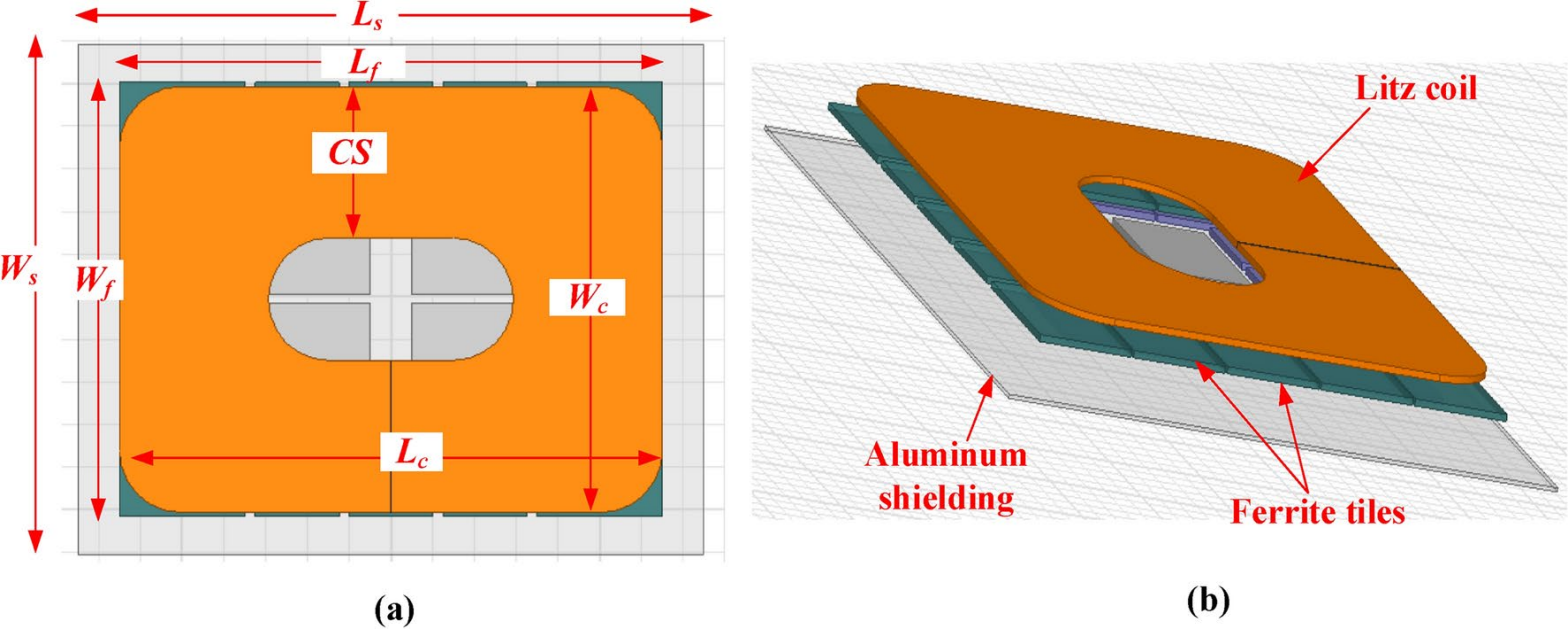
Magnetic modeling of the transmitter pad (CirPT) for *P*_*o*_ level of 11.1-kVA, (**a**) top-view, and (**b**) 3D-view.

3D-FEM for CirPT which fit WPT level 3 charging system and compliant with the Z2-class specifications, is constructed utilizing the ANSYS Maxwell software. The CirPT configuration comprises a two-layer coil crafted from litz wire with a radius of 2.5 mm. The coil windings are represented in the configuration as one block of copper, each representing a single turn, as illustrated in Fig. 2. This designing methodology aids in minimizing computational time and effort for Finite Element Analysis (FEA), while striking a suitable balance between accuracy and model complexity^18^. Furthermore, the CirPT configuration includes three layers of concentrated ferrite (N87), each with a thickness of 5 mm, serving as field lines collectors.

The layers comprise various tiles, with certain tiles measuring 100 mm in length, 100 mm in width, and 5 mm in thickness, while others have dimensions of 100 mm in length, 150 mm in width, and 5 mm in thickness. For the analysis in this paper, the commercial ferrite material N87 is utilized, which is widely employed in IPT configurations. N87 is comprised of a blend of MnZn material, known for its minimized losses at high ranges of frequencies owing to its high permeability. Additionally, its low electrical conductivity aids in minimizing eddy currents. Below the ferrites, an aluminum layer with a height of 3 mm is affixed, positioned at a separation distance of 15 mm. This plate serves as a passive shielding, aiming to diminish the leakage EMFs^19^. The design dimensions of the 3D FEM are listed in Table 2.

**Table 2:** Design dimensions for all configurations at Z2-class.

Para	# Turns	*L*_*c*_ (mm)	*L*_*f*_ (mm)	*L*_*s*_ (mm)	*W*_*c*_ (mm)	*W*_*f*_ (mm)	*W*_*s*_ (mm)	*CS*_*1*_ (mm)	*CS*_*2*_ (mm)
CirPT	16	650	650	750	500	510	600	178.1	–
CirPR	9	320	332.52	350	320	333	350	80	–
DDPR	3 (bifilar)	260	340	390	190	92	270	15.5	31.363

**Fig. 3:**
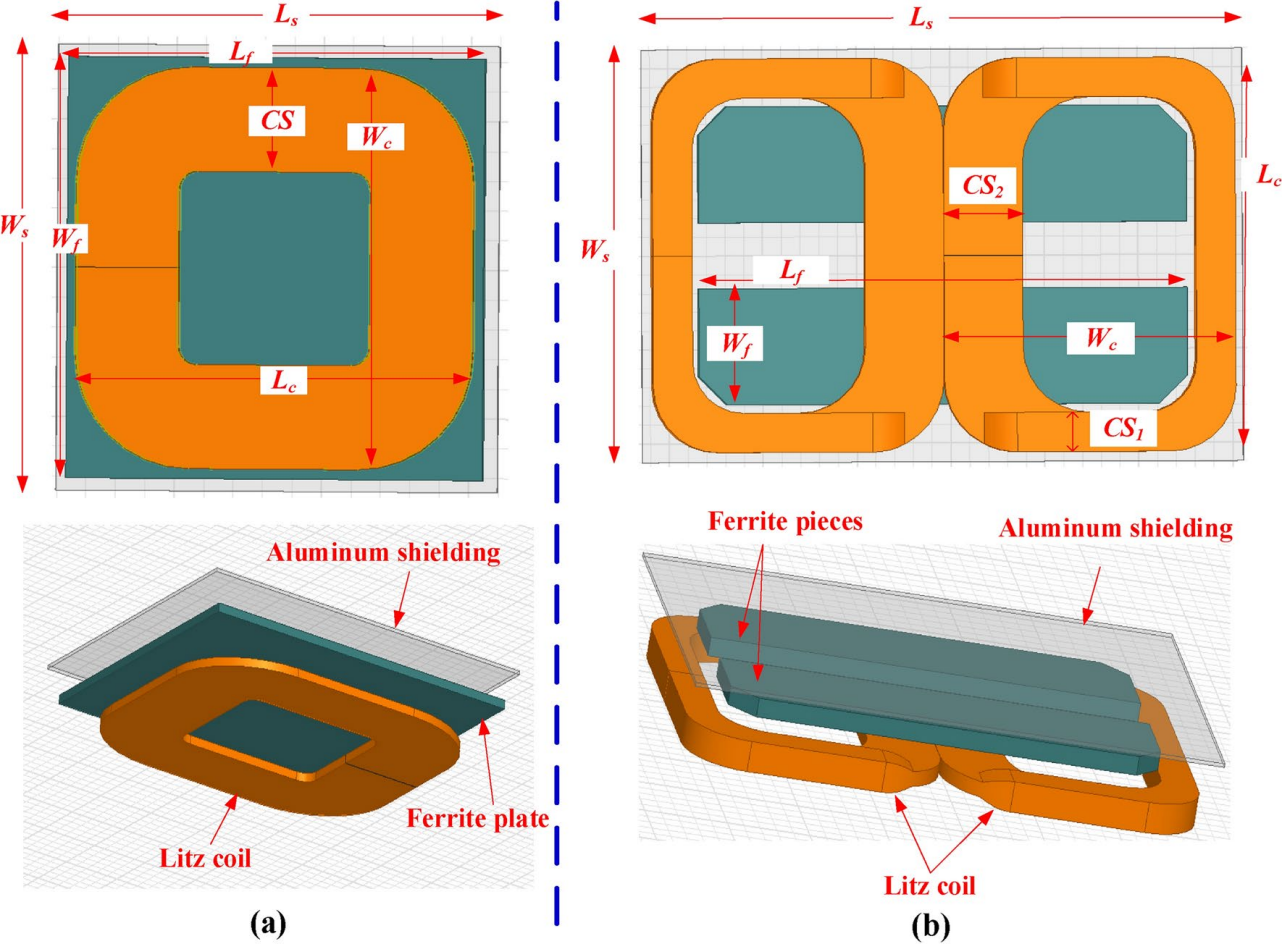
Magnetic modeling of the receiver pads for *P*_*o*_ level of 11.1-kVA, (**a**) CirPR, and (**b**) DDPR.

Much like the CirPT, the CirPR comprises a one-layer coil crafted from litz wire with a 2.5 mm radius. Positioned atop the coil is a ferrite layer with a height of 5 mm, and a separation of 1 mm. Additionally, an aluminum layer with a height of 2 mm is situated on the top of the ferrite layer, with a separation of 5.6 mm, as illustrated in Fig. 3a.

Considering that the GSP is circular in shape, CSP equipped with a DDP must have the capability to charge their batteries. To explore this possibility, DDP configured for WPT level 3 are simulated for the Z2-class, adhering to the guidelines outlined in the SAE J2954. DDP comprises 2-similar rectangular-shaped 2-layer coils crafted using litz wire with a radius of 2.5 mm. These coils are wound in a manner where the windings are positioned adjacent to each other in the central coil sides (CS). However, in the outer CS, every pair of windings are stacked on top of each other, as illustrated in Fig. 3(b). The broader central limbs serve to collect the field lines towards the coil center, while the narrower outer CS aid in minimizing leakage flux. The ferrite layer comprises two elongated bars, each with a thickness of 10 mm, positioned with a separation of 35 mm. These bars are situated overhead the coil, with a separation of 1.6 mm. Additionally, an aluminum layer with a height of 2 mm is incorporated overhead the core, with a separation of 0.4 mm. The dimensions of the two CSP configuration are given in Table 2.

### Estimation of magnetic charger parameters

**Fig. 4:**
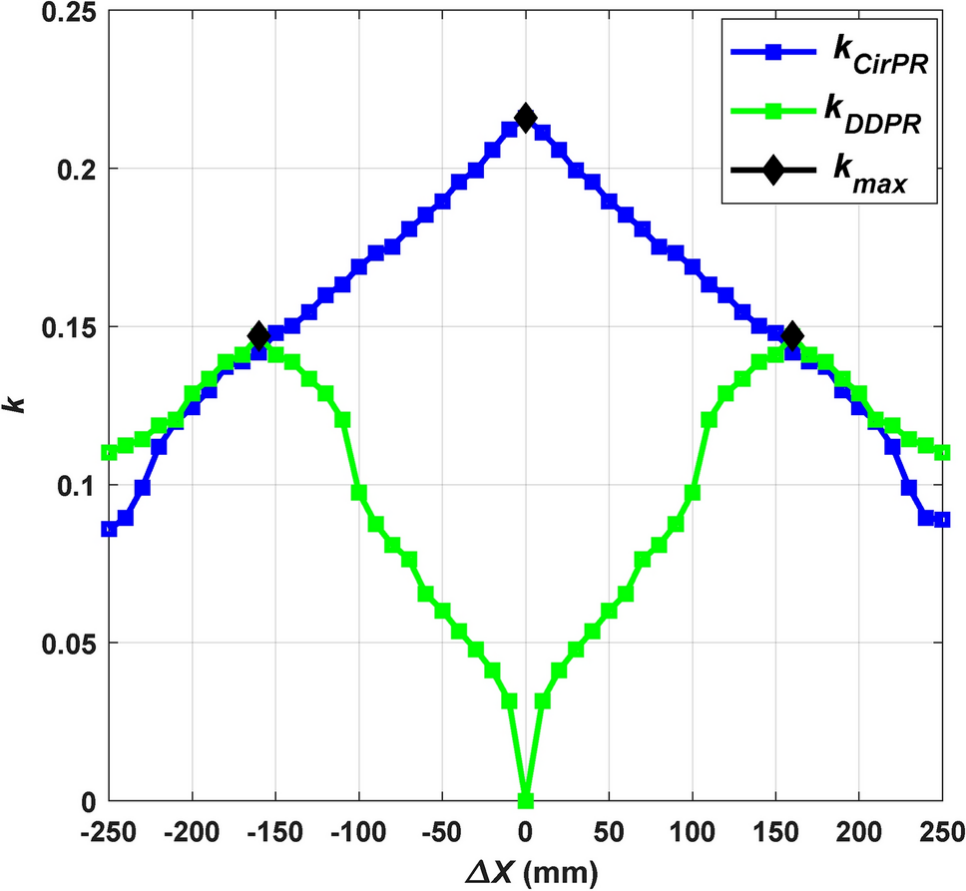
Locations of the maximum values of *k* for the two models at 11.1-kVA along driving direction.

The CirPT is assessed with two configurations of CSP, namely CirPT-CirPR and CirPT-DDPR. The magnetic parameters {GSP inductance (*L*_*p*_), CSP inductance (*L*_*s*_), and coupling factor between GSP and CSP (*k*)} are derived under conditions that yield the maximum *k*, adhering to the maximum limit specified by the Z2-class (airgap = 210 mm). This analysis assumes a Flush ground surface installation, where the airgap equals the ground clearance (see Table 1). The *k* is examined as a function of the driving direction, representing misalignment in the X-direction (*∆X*), for both the CirPT-CirPR and CirPT-DDPR configurations, as illustrated in Fig. 4. The misalignment (Mis) range spans between -25 cm and 25 cm, covering the full spectrum of misalignment in relation to the GSP. As illustrated, the CirPT-CirPR configuration exhibits its highest *k* when the GSP and CSP are ideally aligned. Conversely, for the CirPT-DDPR configuration, the *k* is 0 at ideal alignment and raises progressively with the driving direction. The maximum *k* is reached when the DDPR aligns with the CS of the CirPT. X_1_, Y_1_, Z_1_ is a new coordinate system that has been devised to indicate the location of the maximum *k* for the CirPT-DDPR configuration. These coordinates are shifted from the X, Y, Z by a separation of *∆X* =  ± 160 mm.

The CirPT-CirPR and CirPT-DDPR configurations are examined, and their outcomes are juxtaposed with the range specified in J2954, as outlined in Table 3. The numerical values from both configurations align with the recommended range provided in J2954. In the findings of the CirPT-DDPR configuration, the self-inductances demonstrate strong correlations. Nevertheless, the *k* falls outside the range specified by J2954, which is anticipated when both the GSP and CSP are different. However, the *k* remains within the expected range.

**Table 3:** Comparison of *L*_*p*_, *L*_*s*_, and *k* for the two configurations with the given range within the SAE j2954.

	CirPT-CirPR		CirPT-DDPR	
	Design	J2954-range	Design	J2954-range
${L}_{p}$*(uH)*	37.90	35.10–38.10	37.50	35.10–38.10
${L}_{s}$*(uH)*	44.50	43.50–44.60	13.90	13.60–14.50
$k$	0.216	0.09–0.221	0.147	0.151–0.354

The operation of an IPT configuration at HF, coupled with resonance, results in the coils equivalent series resistance (CESR) exerting a considerable influence on the performance, particularly *η* and quality factor (*Q*). The resistances for both the GSP and CSP are computed using the details outlined in J2954 regarding the coil turns and dimensions. This information is cross-referenced with the datasheet from a manufacturer of Litz wire^20^. Based on the coil specifications provided in J2954, the length of the coils is estimated. Taking into account the *f* range from 79 to 90 kHz and the specified external diameter of the wires in J2954, the DC resistance/length (*R*_*dcl*_) is computed utilizing the datasheet^21^. The alternating current AC resistance (*R*_*ac*_), which considers both the skin and proximity effects, is subsequently computed utilizing Eq. (1)^22^.

1 $$\frac{{R}_{ac} }{{R}_{dcl}}= W+H {\left(\frac{N{D}_{1}}{{D}_{o}}\right)}^{2} {\left(\frac{{D}_{1}\sqrt{f} }{10.44}\right)}^{4}$$

In the equation, *D*_*1*_ signifies the diameter of a strand, *W* represents the resistance ratio of a single strand when isolated, *N* is the number of strands, *D*_*O*_ represents the diameter equivalent to the wire enclosing the strands, and *H* is a constant correlating to *N*, as specified in the datasheet. The calculated CESRs for the three coils, namely CirPT, CirPR, and DDPR, are 0.0437 Ω, 0.01364 Ω, and 0.01658 Ω, respectively.

## Compensation network components (CNCs)

CNCs are employed in IPT configurations to offset the considerable leakage inductance resulting from a substantial airgap, thereby improving the system’s ability to transfer power and its overall efficiency. Furthermore, CNCs contribute to lowering the apparent power (*S*) drawn from the supply by furnishing reactive power and achieving power factor equal to 1 for the operation, facilitating the electronic elements to work at soft switching. CNCs configurations range from utilizing a one capacitor connected in parallel or in series like S–S, S-P, P-S, and P-P^23–26^, to employing combinations of LC circuits like L-C–C, C–C-L, and L-C-L^27–29^. The J2954 guidelines suggest specific CNCs for each pad architecture, which will be elaborated and examined in the following section.

### CNCs for CirPT-CirPR configuration and CirPT-DDPR configuration

**Fig. 5:**
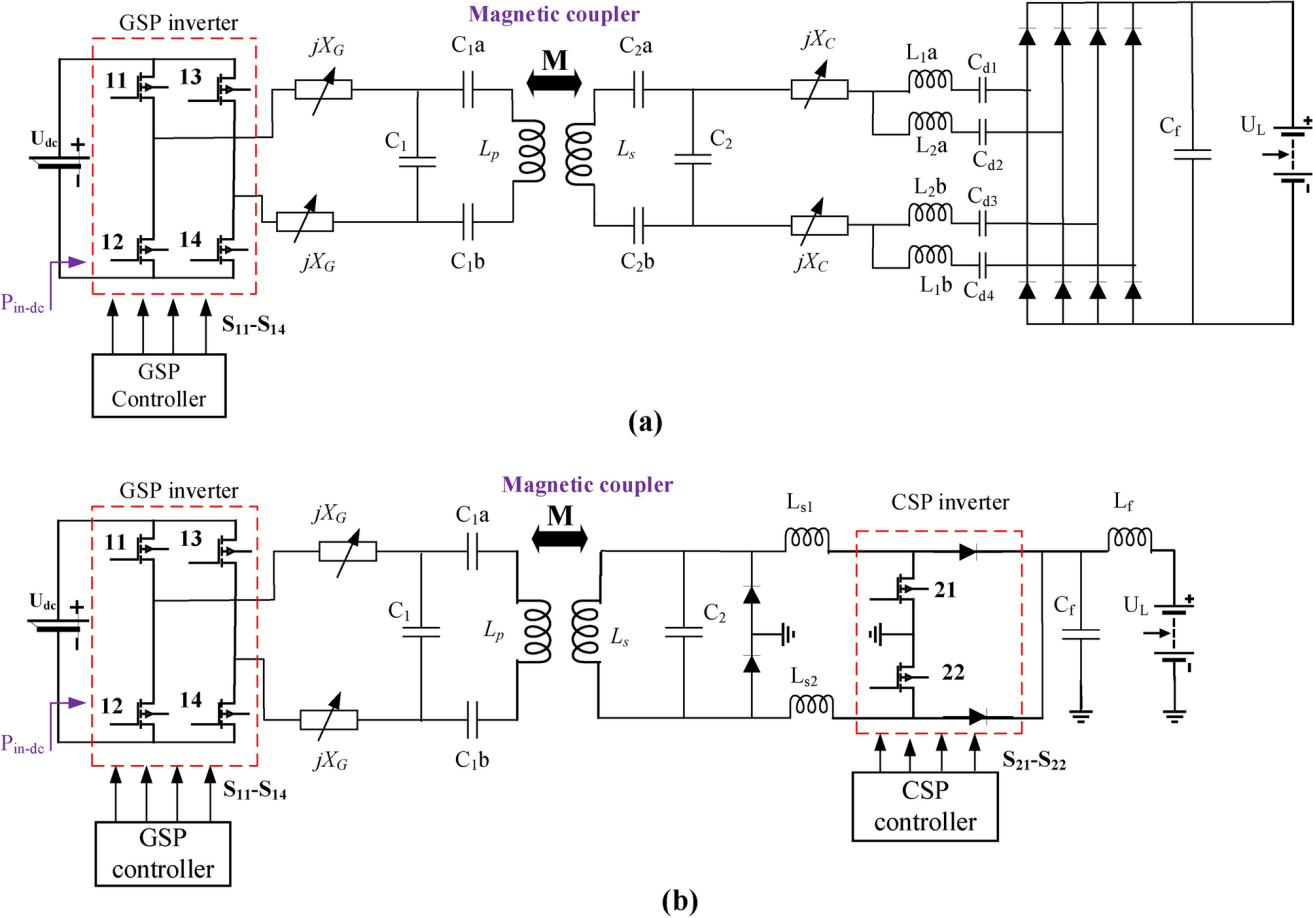
Simulation circuit of WPT level 3/Z2-class configurations, (a) CirPT-CirPR, and (b) CirPT-DDPR.

In the CirPT-CirPR scenario, an LCCL compensation technique is utilized on both the GSP and the CSP, as illustrated in Fig. 5a. The advantages of LCCL compensation circuits in both sides of inductive charging systems are notable for their ability to enhance system efficiency and performance including: (a) the LCCL compensation topology improves misalignment tolerance, achieving a stable output even under significant coil misalignment, which is crucial for practical WPT systems. The circuit maintains high efficiency (above 90%) and minimizes fluctuations in load current during misalignment^30^. (b) similar to LCC circuits, LCCL compensation supports both constant voltage and constant current modes, which are essential for battery charging applications. It effectively handles variations in transmission distances while maintaining a stable output^31^. (c) LCCL circuits are designed to achieve zero-voltage switching (ZVS), which reduces switching losses and enhances overall system efficiency^32^. (d) the integration of LCCL compensation circuits results in more compact systems with fewer components, reducing the overall system size while still maintaining high performance. This is particularly useful in applications like EVs charging, where space is a constraint^33^. These merits make LCCL compensation circuits a strong choice for improving the performance and reliability of inductive charging systems, especially in applications requiring misalignment tolerance and high efficiency as in this manuscript. Additionally, parallel compensation circuits on the receiver side of inductive charging systems offer several advantages including: (a) parallel compensation achieves higher efficiency when there is significant coil misalignment. This makes it especially suitable for EVs charging, where alignment can vary^34^. (b) parallel compensation enables a faster system response while keeping the control input within bounds. This dynamic response capability is useful in WPT systems that require fast adjustments to changing load conditions^35^. (c) parallel compensation circuits ensure the asymptotic stability of the system, which is crucial for maintaining consistent performance and avoiding instability in the power transfer process^35^. These merits highlight why parallel compensation circuits are often preferred for receiver-side design in IPT systems, especially in applications requiring high efficiency and stability under varying conditions.

Although Table 4 offers precise values for most circuit elements, the filter reactance values for both the GSP (*X*_*G*_) and the CSP (*X*_*C*_) are presented as ranges. It’s essential to analyze this reactance within the provided ranges to ensure that the system can realize the anticipated power (11.1 kVA). For *X*_*G*_, a + Ve range of 4 Ω to 16 Ω indicates an inductive reactance, that can be expressed as an inductive value. (*L*_*G*_). Conversely, for *X*_*C*_, the range of -15 to 0 Ω signifies a capacitive reactance, that can be expressed as a capacitance value (*C*_*C*_).

**Table 4:** Electrical requirements of WPT level 3/Z2-class CirPT-CirPR and CirPT-DDPR configurations.

Para	*U*_*dc*_ (V)	*C*_*1*_ (nF)	*C*_*1a*_*, C*_*1b*_ (nF)	*C*_*2a*_*, C*_*2b*_ (nF)	*C*_*2*_ (nF)	*L*_*1a*_*, L*_*1b*_*, L*_*2a*_*, L*_*2b*_ (µH)	*L*_*s1*_*, L*_*s2*_ (µH)	*C*_*d1*_*, C*_*d4*_ (nF)	*C*_*d2*_*, C*_*d3*_ (nF)	*C*_*f*_ (µF)	*L*_*f*_ (µH)	*U*_*L*_ (V)	*X*_*G*_ (Ω)	*X*_*C*_ (Ω)
CirPT-CirPR	500	268	307	284	134	54	–	100	48	30	–	400	4 − 16	−15 − 0
CirPT-DDPR	500	268	307	–	258	–	250	–	–	40	2	320	4 − 16	–

A Simulation model has been constructed and employed to compute the efficiency (*η*) of the two configurations that are illustrated in Fig. 5. This model comprises a DC supply to mimic the utility DC-bus, a MOSFET-based HF inverter, reactance’s filters, CNCs on both charging sides, a magnetic coupler coils, and a diode rectifier on the CSP. By calculating both the input power of the inverter (*P*_*in-dc*_) and the output power at the load (*P*_*L*_), the *η* can be determined through the application of Eq. (2).

2 $${\eta }_{ }=\frac{{ P}_{L} }{{ P}_{in-dc} }$$

The Simulation model undergoes analysis to determine the system’s *f*, GSP filter inductance (*L*_*G*_), and CSP filter capacitance (*C*_*C*_). These elements are specified to guarantee that the configuration transports the nominal power under ideal alignment and achieves the greatest *η*. For this analysis, a *f* from 79 to 90 kHz is taken into account, in accordance with the recommendation provided in J2954.

**Fig. 6:**
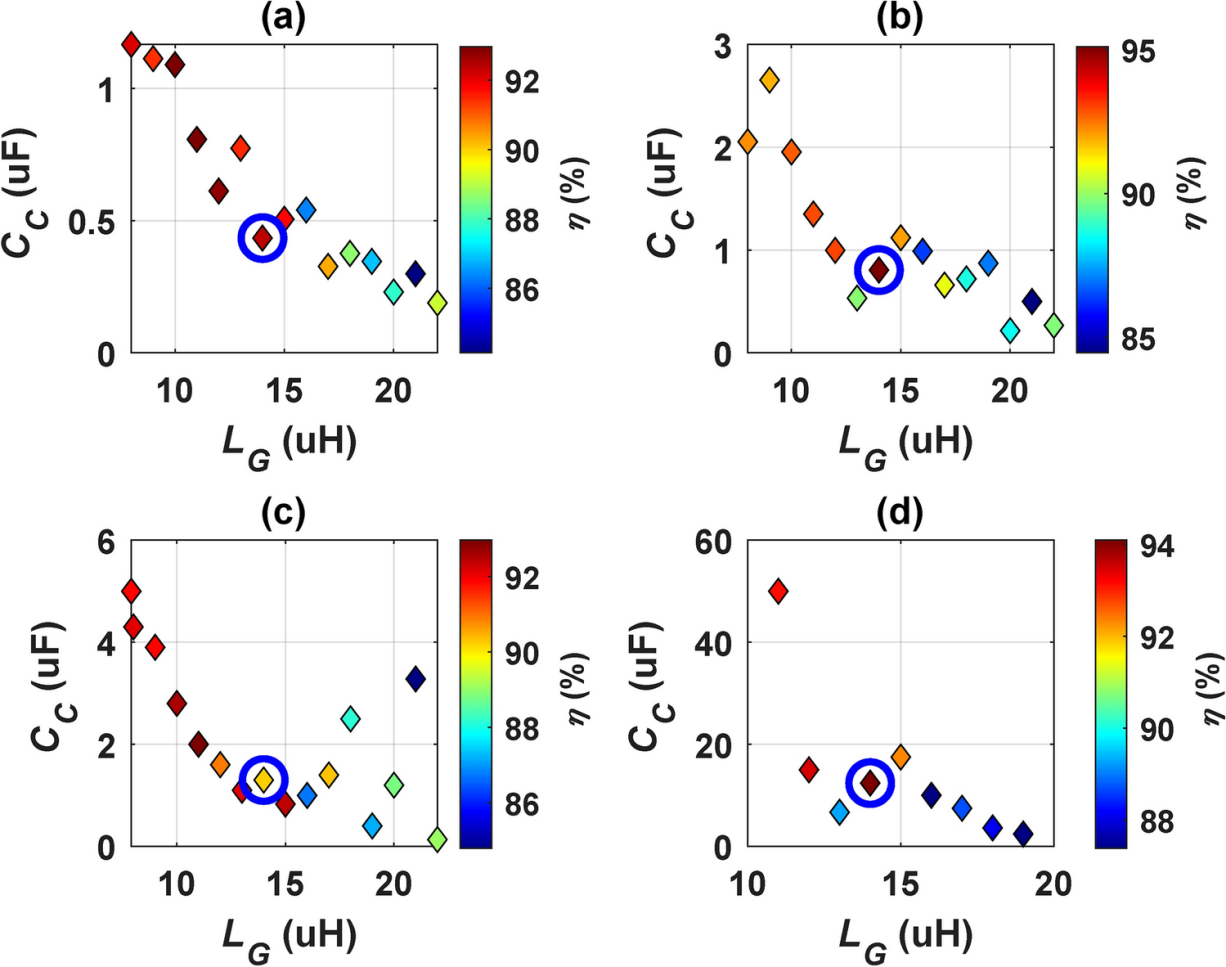
The correlation among, *L*_*G*_, and *η C*_*C*_ for CirPT-CirPR configuration: (**a**) @ *f* equal to 79-kHz, (**b**) @ *f* equal to 79.5-kHz, (**c**) @ *f* equal to 80-kHz, and (**d**) @* f* equal to 80.5-kHz.

In the context of the CirPT-CirPR circuit, Fig. 6 illustrates the correlation between the *f*, GSP filter inductance (*L*_*G*_), CSP filter capacitance (*C*_*C*_), and *η*. At each frequency, *L*_*G*_ is varied within the provided range in steps of 1 µH. For each *L*_*G*_ value, *C*_*C*_ is adjusted until the system achieves the transfer of nominal power. The process involves documenting the corresponding *C*_*C*_ value alongside the *η*, repeating this procedure across the whole range of *L*_*G*_ and operating frequencies. The outcomes at 4 distinct frequencies which are 80.5 kHz, 80 kHz, 79.5 kHz, and 79 kHz presented in Fig. 6, demonstrating the system’s capability to transport the nominal power consistently. Within each plot representing a frequency, the point showcasing the highest *η* is highlighted in blue, and the corresponding elements are tabulated in Table 5. Notably, the CirPT-CirPR configuration achieves its peak *η* (95.1%) at a *f* of 79.5 kHz, with *L*_*G*_ set at 14 µH and *C*_*C*_ at 0.8049 µF.

**Table 5:** Parameters yielding the maximum *η* at each frequency for the CirPT-CirPR configuration.

*f*	79 kHz	79.5 kHz	80 kHz	80.5 kHz
*L*_*G*_	14 µH	14 µH	14 µH	14 µH
*C*_*C*_	0.434 µF	0.8049 µF	1.3 µF	12.4 µF
*η*	92.39%	95.1%	92.15%	94.09%

**Fig. 7:**
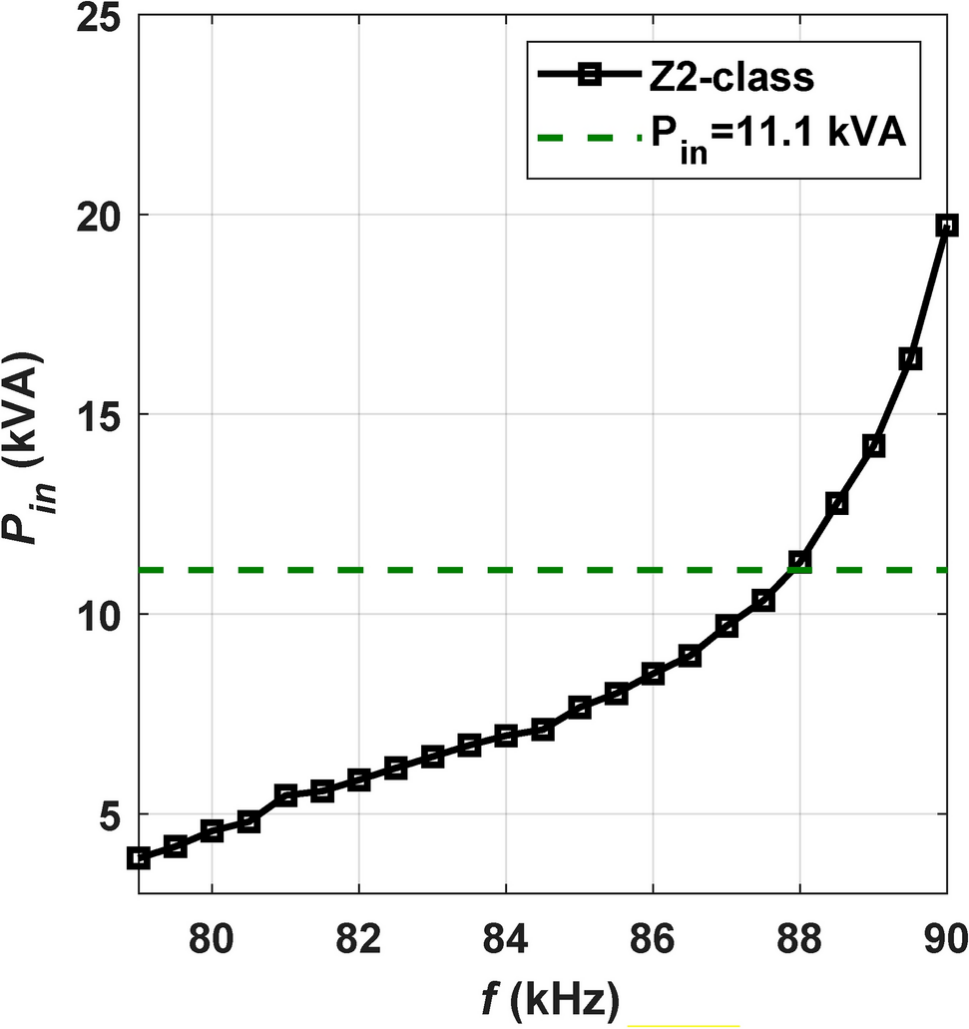
Relationship of *f* and *P*_*in*_ for the CirPT-DDPR configuration.

In the scenario of CirPT-DDPR WPT level 3/Z2-class, the identical GSP circuit is utilized, whereas CSP circuits of DD coil are employed. Therefore, the only variable is the *f*. For DDPR, the J2954 guidelines advocate the implementation of an LC parallel compensation technique, as illustrated in Fig. 5b. The simulation circuit components are detailed in Table 4. To determine the suitable *f*, a Simulation model is constructed and assessed. Figure 7 illustrates the correlation between the *f* and input power (*P*_*in*_) for WPT level3/Z2-class. As illustrated, elevating the *f* enhances the capacity of transported power. Frequencies corresponding to the *P*_*in*_ of 11.1 kVA are identified from the Figure. For Z2-class, operating points with the greatest value of *η* are observed at *f* = 87.879 kHz, achieving an *η* of 91.6%. In the CirPT-DDPR configuration, in order to transfer the equivalent power, the system must operate at HF. This results in reduce the overall *η* compared to that of the CirPT-CirPR configuration.

## Interoperability assessment

Under the assumption that cars may have varying car side pads (CSPs), and future charging ports might feature diverse ground side pads (GSPs), it’s imperative that these GSPs and CSPs seamlessly and effectively interface with one another. This interoperability concept ensures that any car can utilize any charging port, streamlining the charging process. This flexibility eliminates the need for drivers to be restricted to a specific coil type in their car, allowing them to charge from any public port^9,10^. When both GSP and CSP incorporate identical pads kinds, the magnetic flux lines follow the correct trajectory, enabling the charging configuration to efficiently transfer the necessary power. However, if they consist of various pad kinds, the operational dynamics of the system become uncertain, necessitating further investigation. This study focuses on examining the interoperability and misalignment analysis between the two commonly utilized pads (CirP and DDP) suggested by the SAE J2954. The section discusses the performance factors utilized to evaluate interoperability and misalignments.

### Transferred power

In IPT configurations, the term "uncompensated power (*P*_*US*_)" refers to the nominal transported power from a GSP to a CSP without considering any CNCs. It’s calculated by multiplying the short circuit current (*I*_*sc*_) of the CSP coil by the open circuit voltage (*V*_*oc*_), as described in Eq. (3)^36^. The *P*_*US*_ depends on factors such as the current flowing through the GSP coil (*I*_*p*_), the angular operating frequency (*ω*), the mutual inductance (*M*), and the self-inductance of the CSP coil (*L*_*s*_). The actual *P*_*o*_ is influenced by the characteristics of the CSP circuit and the type of CNCs it employs. These factors are determined by the CSP circuit’s quality factor (*Q*_*s*_), as outlined in Eq. (4)^36^.

3 $$\left\{\begin{array}{c}{V}_{oc}=j\omega .M.{I}_{p }\\ {I}_{sc}=\frac{M{I}_{p}}{{L}_{s}} \\ {P}_{us}={V}_{oc} {I}_{sc}=\omega .{{I}_{p}}^{2}.\frac{{M}^{2}}{{L}_{s}}\end{array}\right.$$

4 $${P}_{o}= \omega .{{I}_{p}}^{2}.\frac{{M}^{2}}{{L}_{s}}.{Q}_{s}$$

The *P*_*o*_ can also be represented using *Q*_*s*_, the *k*, and the Volt-Ampere rating of the GSP circuit (*VA*_*p*_), as detailed in Eq. (5)^36^. A substantial increase in the *k* significantly enhances the capability of power transfer. This factor is contingent upon the self-inductances of the GSP and CSP coils (*L*_*p*_ and *L*_*s*_) as well as the mutual inductance between them (*M*), as described in Eq. (6).

5 $${P}_{out} = {V}_{p }{I}_{p} {k}^{2}{Q}_{s}={VA}_{p}{ k}^{2}{Q}_{s}$$

6 $$k=\frac{M}{\sqrt{{L}_{p} {L}_{s}}}$$

To delve into the study of misalignments and interoperability, the initial prerequisite is the ability to consistently transfer the nominal power under various conditions. When considering a CirP as a universal GSP coil, the charging pad must deliver the necessary power to a car irrespective of its ground clearance and whether it incorporates CirP or DDP configuration.

Another essential criterion for interoperable operation is the system’s efficiency in power transfer. The maximum *η* of an IPT system correlates directly with the *k* and *Q* of both the GSP and CSP circuits, as illustrated in Eq. (7)^37^.

7 $${\eta }_{max }=\frac{k \sqrt{{Q}_{L1}{Q}_{L2}}}{2+k \sqrt{{Q}_{L1}{Q}_{L2}}}$$

### Misalignment scenarios

Due to the inability of cars to be parked precisely overhead charging pads, an IPT configuration needs to accommodate a variety of offsets in various directions to accommodate anticipated misalignments in real-world usage. The system is anticipated to encounter linear offsets in the X-axis (ΔX), Y-axis (ΔY), and Z-axis (ΔZ). Furthermore, angular offsets may also occur, known as *Yaw *^o^, *Pitch *^o^, and* Roll *^o^. The J2954 standard has established acceptable ranges for these misalignments, detailed in Table 6, which are considered in this investigation.

**Table 6:** IPT Misalignment scenarios.

Misalignment	Ranges
*∆Z* (mm)	100 − 150 for Z1 140 − 210 for Z2 170 − 250 for Z3
*∆X* (mm)	± 75
*∆Y* (mm)	± 100
*Yaw* °	± 10
*Pitch* °	± 2
*Roll* °	± 2

## Discussion and findings

The examination of the developed FEMs in conjunction with simulated models for CirPT-CirPR and CirPT-DDPR configurations aims to investigate the interoperability and misalignments between the universal GSP coil and various CSP coils. The configurations performance is assessed under various misalignment conditions, specifically considering the WPT level 3/Z2-class. In CirPT-CirPR configuration, misalignments are evaluated based on X–Y-Z coordinates, while for CirPT-DDPR configuration, they are gauged in relation to the coordinates X_1_-Y_1_-Z_1_, accounting for the inherent offset.

### Simulated models performance

**Fig. 8:**
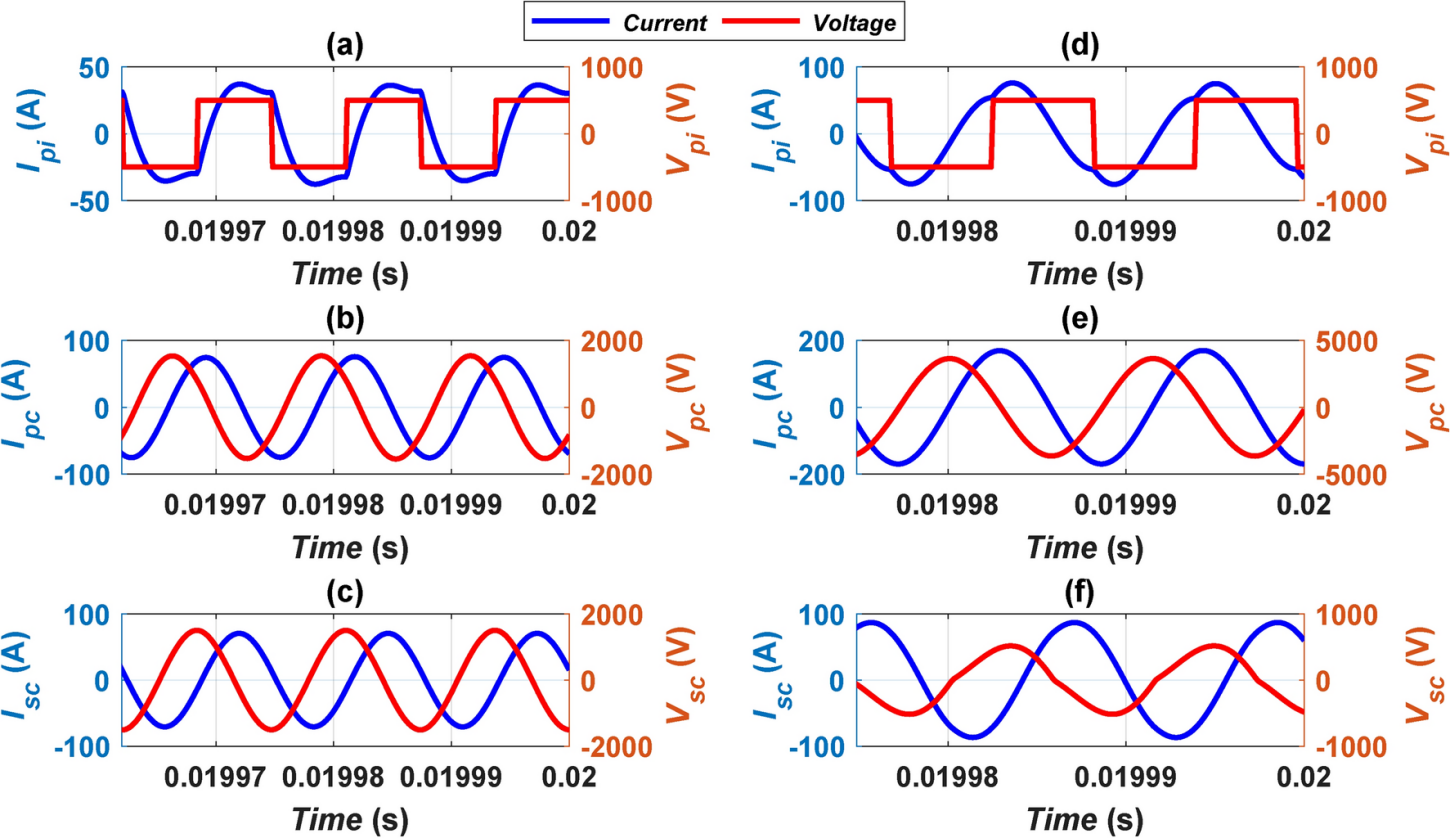
Simulated performance for Z2-class model: (**a**)* I*_*pi*_ and *V*_*pi*_ for CirPT-CirPR configuration, (**b**)* I*_*pc*_ and *V*_*pc*_ for CirPT-CirPR configuration, (**c**)* I*_*sc*_ and *V*_*sc*_ for CirPT-CirPR configuration, (**d**)* I*_*pi*_ and *V*_*pi*_ for CirPT-DDPR configuration, (**e**)* I*_*pc*_ and *V*_*pc*_ for CirPT-DDPR configuration, and (**f**)* I*_*sc*_ and *V*_*sc*_ for CirPT-DDPR configuration.

This section delves into an examination of the electric circuit models pertaining to both CirPT-CirPR and CirPT-DDPR configurations. The parameters linked with the Z2-class at ideal alignment conditions are taken into account for this analysis. Figure 8 depicts and compares the output voltage and current waveforms for the GSP inverter (*V*_*pi*_, *I*_*pi*_), GSP coil (*V*_*pc*_, *I*_*pc*_), and CSP coil (*V*_*sc*_, *I*_*sc*_) respectively, for both CirPT-CirPR and CirPT-DDPR configurations under the Z2-class conditions. The GSP inverter generates a square-wave voltage waveform and a semi-sinusoidal current shape, with some noticeable harmonics, as depicted in Fig. 8a and d respectively. These waveforms undergo filtration via the LCCL compensation technique, resulting in a nearly sinusoidal shape observed in the GSP coil, as illustrated in Fig. 8b and e. The sinusoidal current flowing through the GSP coil induces sinusoidal voltage and current waveforms at the identical frequency within the CSP coil circuit.

In the CirPT-DDPR model, the CSP coil voltage exhibits some harmonic content owing to the presence of the parallel compensation capacitor in the CSP circuit. In theory, the phase angle between the GSP and CSP coil currents is expected to be equal to 90 degrees. Nevertheless, variations from this theoretical value may arise based on the compensation circuit type, as documented in^38^. In this investigation, a regular 90-degree phase angle is achieved for the CirPT-CirPR configurations. Nevertheless, the CirPT-DDPR configurations exhibit a phase angle > 90 degrees, as depicted in Fig. 8.

### CirPT, CirPR, and DDPR configurations performance

**Fig. 9:**
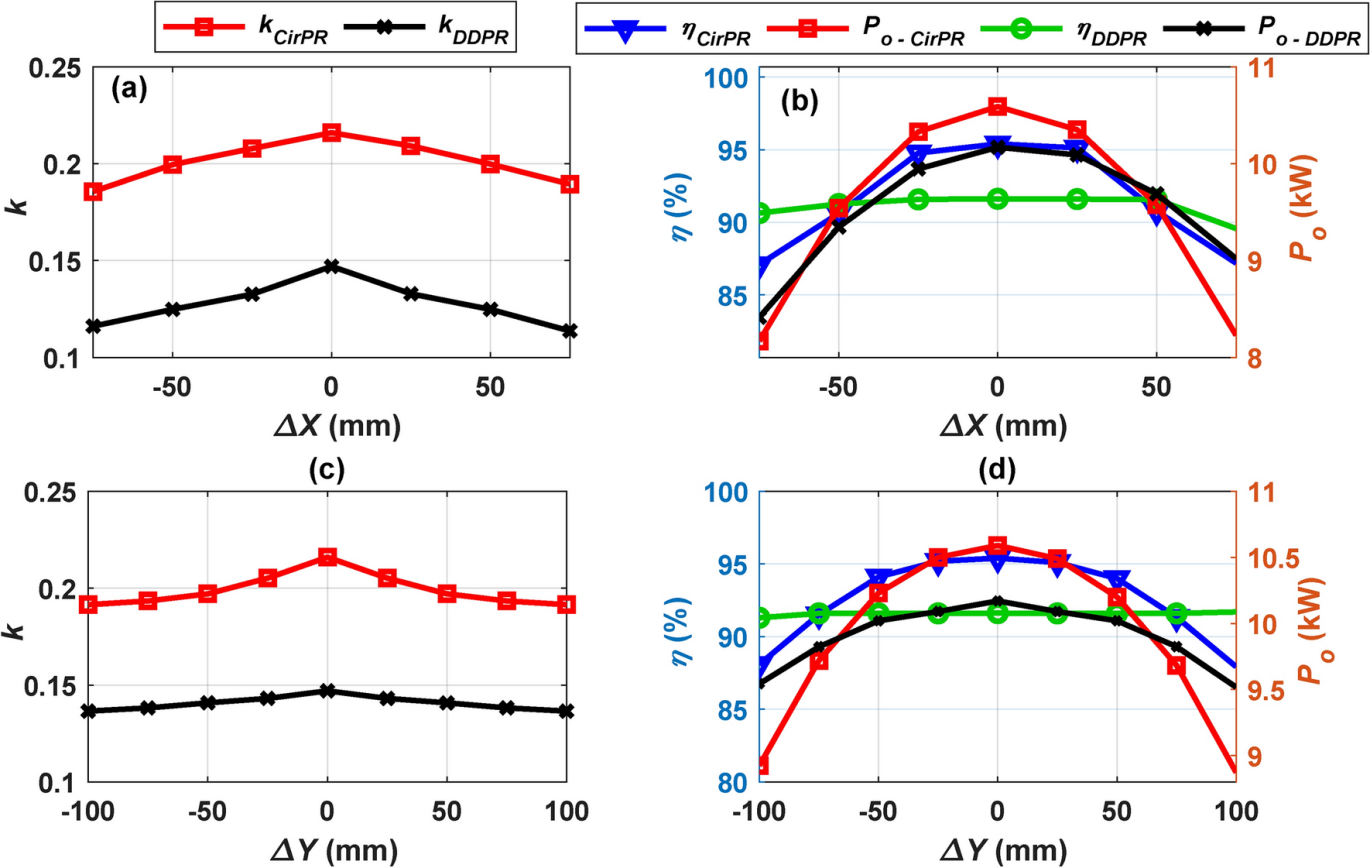
Performance of CirPT-CirPR and CirPT-DDPR configurations for lateral misalignments, (**a**) *k* vs. *∆X*, (**b**) *η, P*_*o*_ vs. *∆X*, (**c**) *k* vs. *∆Y*, and (**d**) *η, P*_*o*_ vs. *∆Y*.

This section investigates the performance of both CirPT-CirPR and CirPT-DDPR configurations under various misalignment scenarios. For each configuration, the *k*, *P*_*o*_, and *η* are evaluated and compared across different degrees of misalignment. The performance of the two configurations under linear misalignments along the X-axis and Y-axis is illustrated in Fig. 9 and summarized in Table 7. In the CirPT-CirPR configuration, zero misalignment aligns with the origin point of the coordinates X–Y-Z, whereas in the CirPT-DDPR configuration, it aligns with the coordinates X_1_-Y_1_-Z_1_. Figure 9a and c demonstrate that the CirPT-CirPR configuration consistently exhibits a higher *k* compared to the CirPT-DDPR configuration across various misalignments in the X- and Y-axis. Furthermore, the CirPT-DDPR configuration demonstrates a more significant decrease in the *k* from ideal alignment to greatest value of misalignment in the X-axis, while exhibiting a lower decrease in the Y- axis compared to the CirPT-CirPR. This behavior of the *k* is mirrored in the *P*_*o*_ and *η* of the two configurations, as illustrated in Fig. 9b and d. Both configurations exhibit resilient performance when subjected to misalignment in the Y-axis, as indicated by the minimal decrease in *P*_*o*_ and *η* from perfect alignment to greatest offset, as shown in Table 7. Despite the CirPT-CirPR demonstrating slightly superior *P*_*o*_ and significantly higher *η* compared to the CirPT-DDPR, both systems’ efficiencies fall within the acceptable limits specified by J2954 (*η* > 85% for ideal alignment && *η* > 80% for misalignments). Due to a substantial decrease in the *k*, both configurations undergo notable declines in *P*_*o*_ and *η* when misaligned in the X-direction. Specifically, for the CirPT-CirPR configuration, the *P*_*o*_ and *η* drop by 23.58% and 8.41% respectively, while for the CirPT-DDPR, the decrease is 17.64% in *P*_*o*_ and 2.29% in *η*. Nevertheless, the *η* remains above the accepted threshold, which falls between 87.1% and 95.10%. It’s noteworthy that the CirPT-CirPR configuration displays greater sensitivity to linear misalignments compared to the CirPT-DDPR configuration.

**Table 7:** *η* and *P*_*o*_ ranges for CirPT-CirPR and CirPT-DDPR under misalignment scenarios at Z2-class.

Alignment case	CirPT-CirPR (*η* = 95.10% and *P*_*o*_ = -10.60 kW)		CirPT-DDPR (*η* = 91.60% and *P*_*o*_ = 10.20-kW)	
Param	*P*_*o,offset*_ (worst misalignment)	*η*_*offset*_ (worst misalignment)	*P*_*o,offset*_ (worst misalignment)	*η*_*offset*_ (worst misalignment)
*∆Y*	8.80 kW	87.90%	9.50 kW	91.30%
*∆X*	8.10 kW	87.10%	8.40 kW	89.50%
*Yaw*	10.40 kW	94.90%	9.80 kW	91.50%
*Pitch*	9.80 kW	92.10%	9.50 kW	91.4 0%
*Roll*	9.80 kW	92.10%	9.80 kW	91.5 0%

**Fig. 10:**
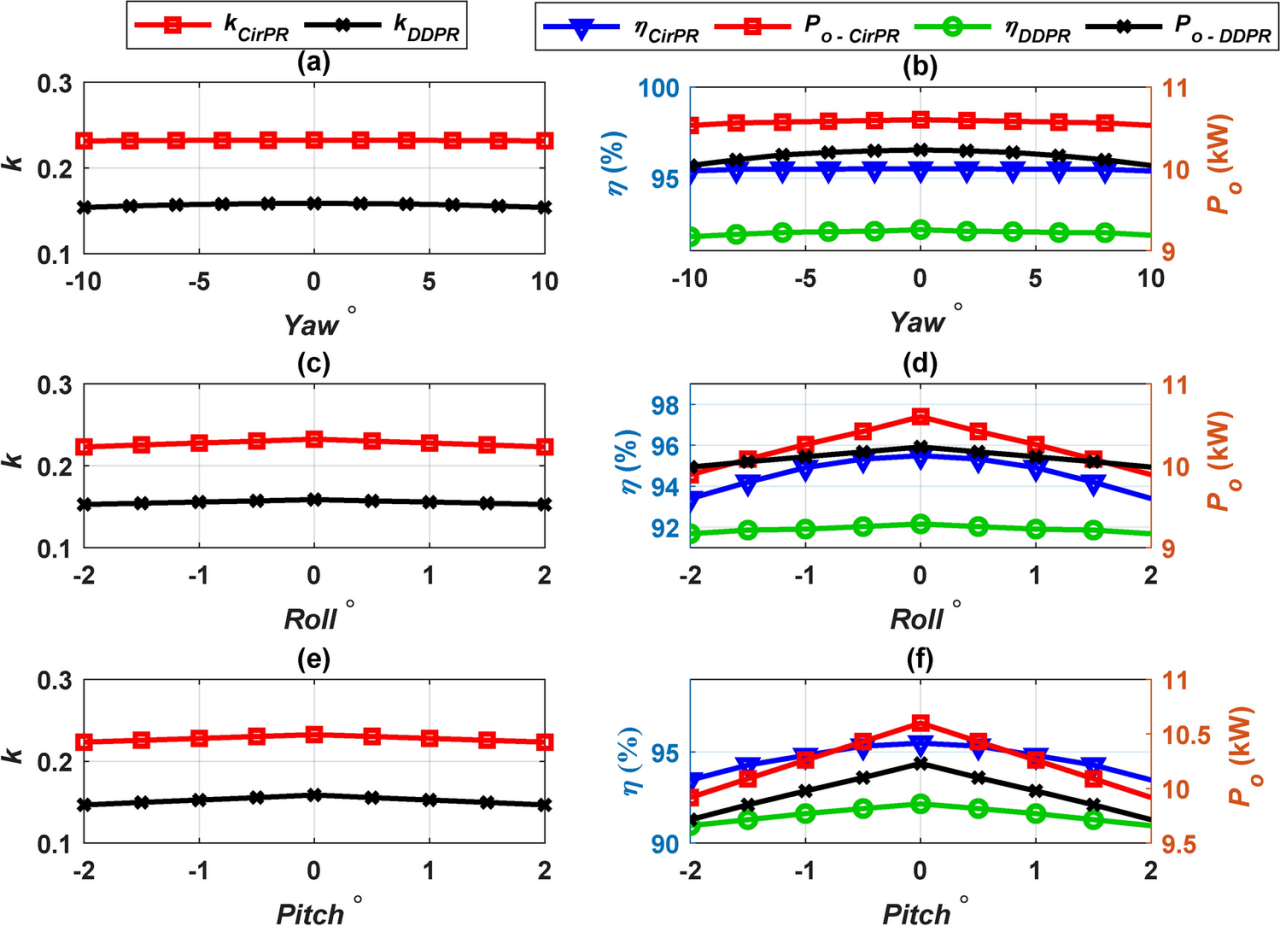
Performance of CirPT-CirPR and CirPT-DDPR configurations for angular misalignments, (**a**) *k* vs. *Yaw*, (**b**) *η, P*_*o*_ vs. *Yaw*, (**c**) *k* vs. *Roll*, (**d**) *η, P*_*o*_ vs. *Roll,* (**e**) *k* vs. *Pitch*, (**f**) *η, P*_*o*_ vs. *Pitch*.

The performance of CirPT-CirPR and CirPT-DDPR configurations under various rotational misalignments (*Pitch *^o^, *Roll*^o^, and *Yaw *^o^) is depicted and contrasted in Fig. 10 and summarized in Table 7. Both configurations exhibit highly similar *k* profiles, indicating small reduction with angular misalignments in comparison to linear misalignments. The CirPT-DDPR configuration demonstrates remarkable resilience to rotational misalignment, as evidenced by consistent *P*_*o*_ and *η* profiles. In contrast, the CirPT-CirPR system exhibits a slight reduction in *P*_*o*_ (1.88% to 7.54%) and *η* (0.21% to 3.15%) with rotational misalignment. However, the *η* remains within the permissible thresholds, ranging from 92.1% to 95.1%, across all angular misalignment conditions.

Based on the aforementioned analysis for Z2-class, it is evident that both CirP and DDP systems on the vehicle side can effectively operate with the universal CirP. Additionally, implementing variable frequency operation to mitigate the effects of misalignment can further enhance performance. As a result, the principle of interoperability is successfully realized, ensuring efficient power transfer in both scenarios.

### Safety assessment

In an IPT system, EMFs are responsible for transferring power from the transmitting coil to the receiving coil. When the transmitter coil is energized, it generates a substantial amount of EMFs that travel across the airgap to the receiver. While some of these fields dissipate into the surrounding air, others engage with the receiving coil to produce useful power. However, if these stray EMFs exceed the safety limits established by international organizations, they can pose a risk to the health of nearby living organisms^39,40^. Various international organizations, including the ICNIRP 2010 guidelines, have established safety limits for EMFs emissions across different frequencies. According to the this guidelines, the recommended safe limits for external magnetic field density (*B*) are 15 µT for pacemakers and 27 µT for living organisms^7,41^. Taking the ICNIRP guidelines into account, the SAE J2954 committee established that 15 µT is the safe general limit for the *B* of the charging system. This value was chosen to ensure the safety of both living organisms and pacemakers. Additionally, the same guidelines specify a safe limit of 83 V/m for electric fields^42^.

**Fig. 11:**
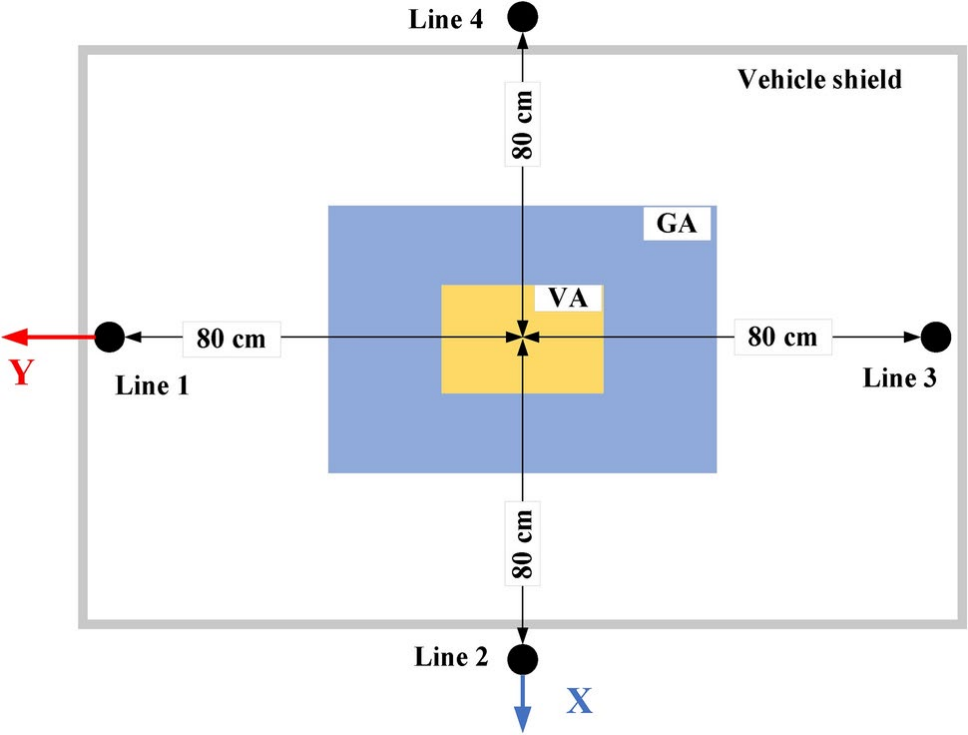
SAE J2954 test points of EMFs.

Since magnetic fields are closely linked to coil currents, various current waveform instances are analyzed, and the one with the highest *B* is selected for magnetic field analysis. *B* is measured along lines 1–4 at the worst air gap distance (210 mm) as shown in Fig. 11; however, due to the symmetry in the CirPT-CirPR model, lines facing each other yield similar results. Therefore, only the results from lines 1 and 2 are presented. In the CirPT-DDPR design, lines 1 and 3 yield very similar results because of the symmetry along the Y1-axis, so only the results from line 1 are shown. Additionally, due to the natural offset (± 160 mm) in the X1-axis, as explained in Section "Estimation of magnetic charger parameters", line 4 exhibits higher EMFs than line 2.

**Fig. 12:**
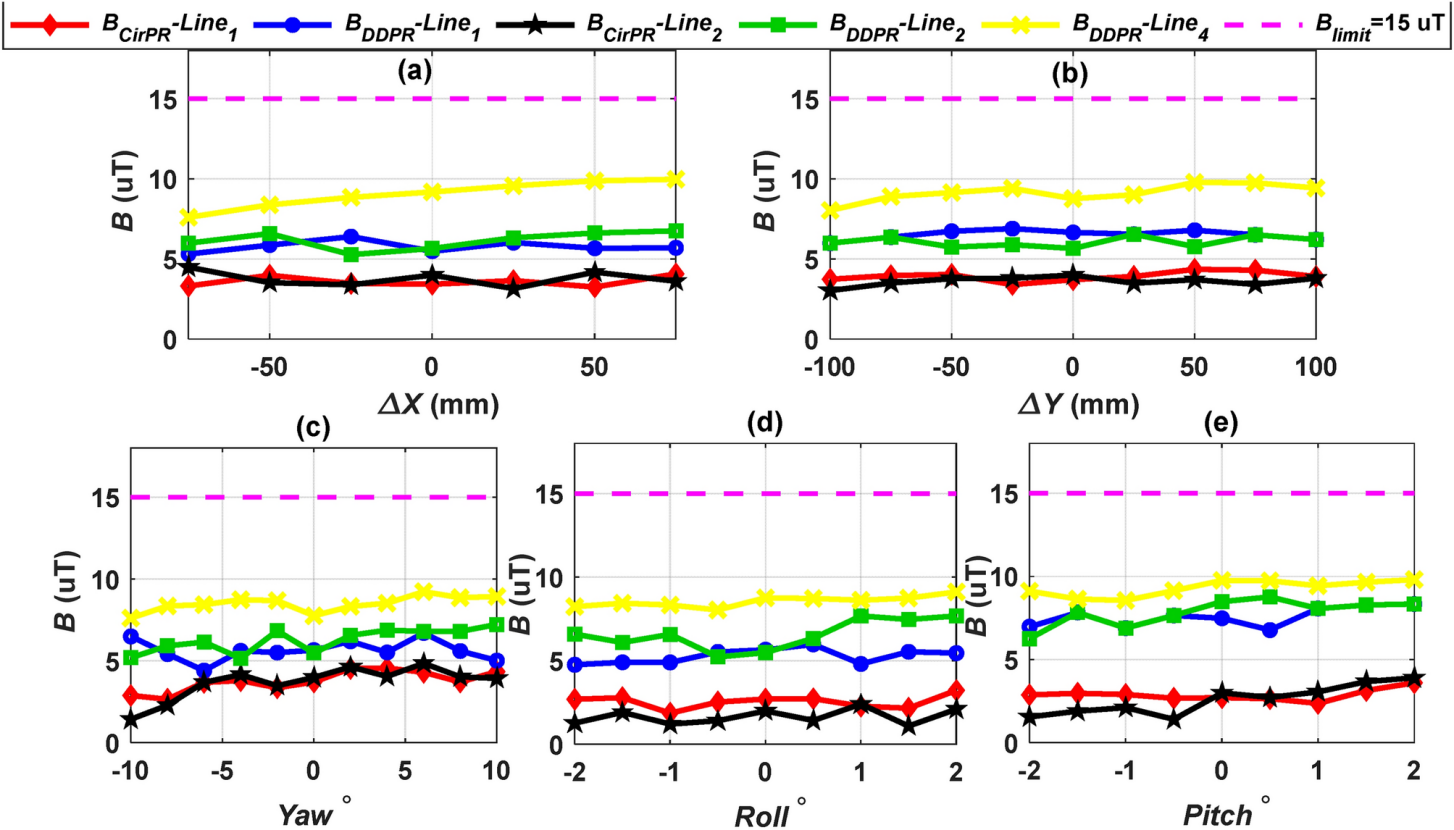
*B* lines distribution for CirPT-CirPR and CirPT-DDPR configurations at the front (line 1), left (line 2), and right (line 4) sides of the EV, (**a**) *B* vs.* ∆X*, (**b**) *B* vs. *∆Y*, (**c**) *B* vs. *Yawº*, (**d**) *B* vs. *Rollº*, and (**e**) *B* vs. *Pitchº*.

Figure 12 displays the measured *B* values for the CirPT-CirPR and CirPT-DDR models across various misalignments. Both systems exhibit leakage magnetic fields that are below the safe limit, maintaining at least a 33.33% margin across different misalignments. This margin accounts for measurement uncertainties, which are usually around 5%^43^. The CirPT-CirPR model exhibits nearly identical *B* levels at lines 1 and 2. In contrast, the CirPT-DDPR system records higher *B* values at all lines across various misalignments, with line 4 reaching the highest values due to the natural offset. Therefore, it can be concluded that both the CirPT-CirPR and CirPT-DDR systems meet the safe limits of ICNIRP 2010 guidelines for *B*.

**Fig. 13:**
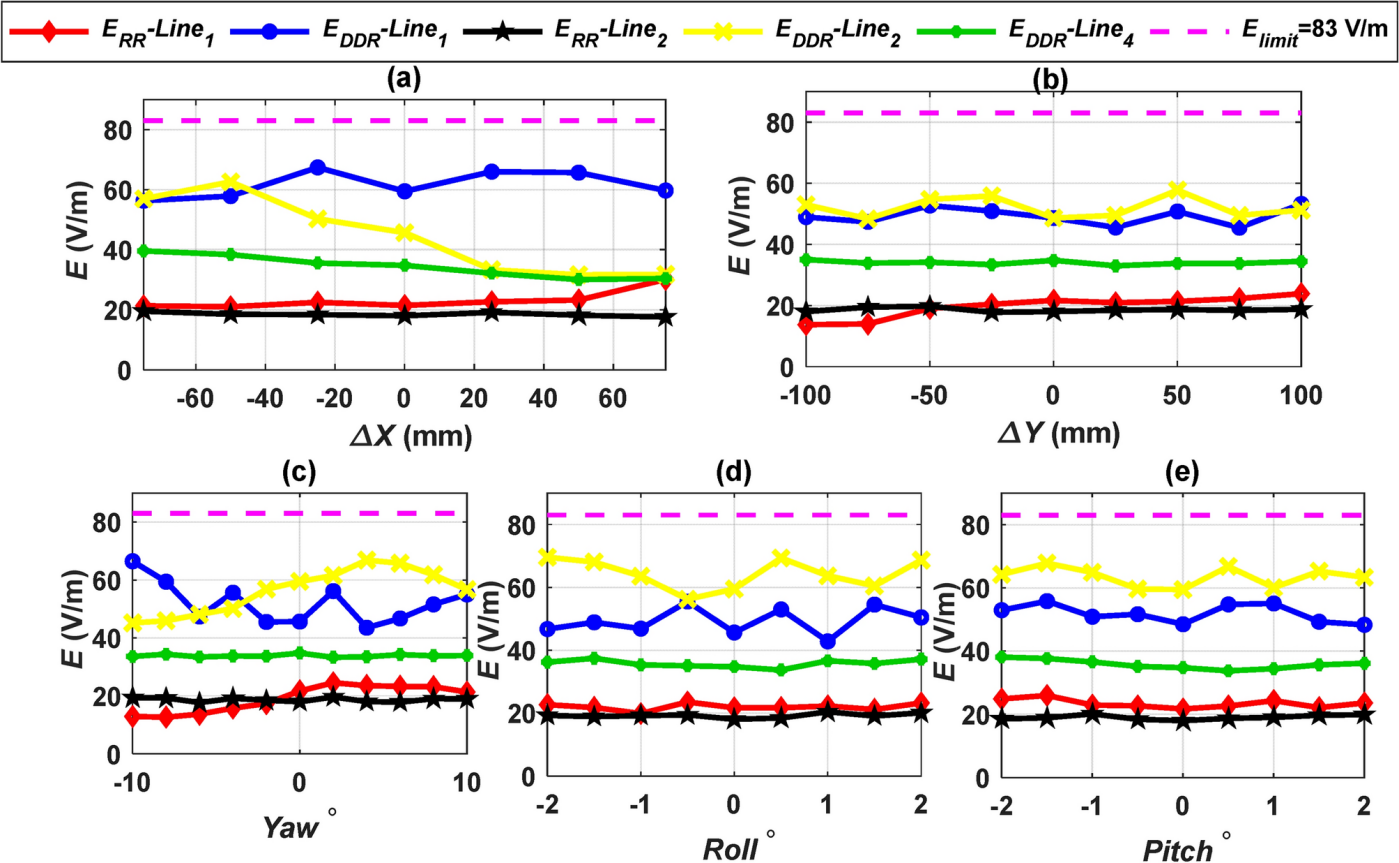
*E* lines distribution for CirPT-CirPR and CirPT-DDPR configurations at the front (line 1), left (line 2), and right (line 4) sides of the EV, (**a**) *E* vs.* ∆X*, (**b**) *E* vs. *∆Y*, (**c**) *E* vs. *Yawº*, (**d**) *E* vs. *Rollº*, and (**e**) *E* vs. *Pitchº*.

In addition to the safe limits for the magnetic field, the electric field around the charging system must also remain within the limits established by the ICNIRP 2010 guidelines. The 2010 guidelines established a maximum safe limit for electric field intensity (*E*) at 83 V/m^42^. The *E* values were measured for each design at the same test lines as the *B* under various linear and angular misalignments. By extracting the voltages from the transmitting and receiving coils in the MATLAB Simulink circuit, the electric field intensity *E* can be estimated. These values are then entered into the corresponding FEMs. Figure 13 shows the *E* values for all configurations at the worst air gap distance (210 mm) and under different misalignment conditions at lines 1, 2, and 4. All *E* values remain below the suggested safe limit of 83 V/m. Therefore, we can conclude that every system presented in this research (CirPT-CirPR and CirPT-DDR) fully complies with the safe limits for electric field *E* as outlined in the ICNIRP 2010 guidelines.

## Conclusion and future work

The paper conducts an extensive investigation to explore the misalignment cases and interoperability between the universal CirPT and both CirPR and DDPR configurations. Specifically focusing on the SAE J2954 specifications for WPT level 3/Z2-class (11.1 kVA), the design of CirPR and DDPR configurations is examined alongside the universal CirPT. The study involves the development, validation, and analysis of circuits and 3D FEMs for both configurations under Z2-class conditions. Parameters such as *f* and CNCs are fine-tuned within the prescribed ranges outlined in the SAE J2954 standard to achieve optimal *P*_*o*_ and maximum *η*. Various performance metrics, including *k*, *P*_*o*_, *η*, and leakage EMFs, are introduced to validate the interoperability and misalignment concepts. These metrics are evaluated across different lateral and angular misalignment scenarios. The study concludes that both CirP and DDP configurations on the CSP can effectively operate with the universal GSP. Additionally, in perfect alignment, the efficiencies are 95.10% for the CirPT-CirPR model and 91.60% for the CirPT-DDPR model. In maximum misalignment, the efficiencies are 87.10% for the CirPT-CirPR model and 89.50% for the CirPT-DDPR model, all exceeding the acceptable threshold of 80%. Furthermore, the implementation of variable frequency operation proves beneficial in mitigating misalignment effects and enhancing overall performance. Consequently, the principle of interoperability is successfully demonstrated, ensuring efficient power transfer across different scenarios.

The analysis and findings of the study suggest several potential research avenues for future investigation, including:

- Carrying out thorough experimental validation to delve deeper into misalignment analysis and interoperable operation for this study.
- Investigating the feasibility of employing DDP as a universal GSP and assessing the interoperability of CirPR and DDPR configurations with experimental validation.
- Investigating the compatibility between a CSP and both static and in-motion GSP, considering the varied installation methods with the way.

## Data Availability

The data used to support the findings of this study are available from the corresponding author upon request.

## Abbreviations

CirPT: Circular pad transmitter
CirPR: Circular pad receiver
DDPR: Double-D pad receiver
DDQP: Double-D quadrature pad
*f*: Frequency
*k*: Coupling coefficient
*P*_*o*_: Transmission power
*η*: Transmission efficiency
EVs: Electric vehicles
IPT: Inductive power transfer
GSP: Ground side pad
CSP: Car side pad
HF: High frequency
CNCs: Compensating network components
NPPs: Non-polarized pads
CirP: Circular pad
RP: Rectangular pad
PPs: Polarized pads
DDP: Double-D pad
BP: Bipolar pad
SP: Solenoid pad
ATs: Ampere-turns
FEMs: Finite-element models
EMFs: Electromagnetic fields
GCR: Ground clearance rage
LDEVs: Light-duty EVs
WPT: Wireless power transfer
SAE: Society of automotive engineering
FEA: Finite Element Analysis
MnZn: Manganese Zinc
CS: Coil sides
*P*_*L*_: Load power
*Q*: Quality factor
*P*_*US*_: Uncompensated power
*L*_*p*_: Transmitter inductance
*L*_*s*_: Receiver inductance
*P*_*in-dc*_: Input power of the inverter
S–S: Series-series
S-P: Series–parallel
P-S: Parallel-series
P-P: Parallel-parallel
CESR: Coils equivalent series resistance
ICNIRP: International Commission on Non-Ionizing Radiation Protection
